# Risk of self-harm ideation in mothers of children with orofacial cleft defects: the Japan environment and children's study

**DOI:** 10.3389/fgwh.2024.1302808

**Published:** 2024-09-17

**Authors:** Shinobu Tsuchiya, Masahiro Tsuchiya, Haruki Momma, Kaoru Igarashi, Ryoichi Nagatomi, Masatoshi Saito, Takahiro Arima, Nobuo Yaegashi, the Japan Environment and Children’s Study, Michihiro Kamijima

**Affiliations:** ^1^ Department of Orthodontics and Speech Therapy for Craniofacial Anomalies, Tohoku University Hospital, Sendai, Japan; ^2^ Division of Oral Dysfunction Science, Tohoku University Graduate School of Dentistry, Sendai, Japan; ^3^ Department of Nursing, Tohoku Fukushi University, Sendai, Japan; ^4^ Department of Medicine and Science in Sports and Exercise, Tohoku University Graduate School of Medicine, Sendai, Japan; ^5^ Division of Biomedical Engineering for Health & Welfare, Tohoku University Graduate School of Biomedical Engineering, Sendai, Japan; ^6^ Department of Obstetrics and Gynecology, Tohoku University Graduate School of Medicine, Sendai, Japan; ^7^ Department of Informative Genetics, Environment and Genome Research Center, Tohoku University Graduate School of Medicine, Sendai, Japan

**Keywords:** cleft lip and palate, congenital anomaly, self-harm ideation, Edinburgh postnatal depression scale, nationwide birth cohort

## Abstract

**Introduction:**

Cleft lip and/or palate (CL/P), the most prevalent congenital anomaly, has been associated with higher rates of child maltreatment. In particular, the presence of cleft lip has more of an impact on external appearance and may increase the risks of negative health outcomes such as parental postpartum depression; however, this concept remains controversial. Item #10 of the Edinburgh Postpartum Depression Scale is the assessment of parental self-harm ideation, and its presence in postpartum mothers merits risk assessments as an emergent issue that may affect the health of both mothers and infants. This study focused on the impact of CL/P on maternal self-harm ideation.

**Methods:**

Of 100,300 live births from a nationwide birth cohort in Japan, 238 mothers of infants with CL/P [186 children born with cleft lip (CL ± P) and 52 born with isolated cleft palate (CP)] were included in the analyses. The prospective association of children with CL/P and maternal self-harm ideation, which were acquired using item #10 in the Edinburgh Postpartum Depression Scale at 1 and 6 months postpartum, was examined using binomial logistic regression analyses after multiple imputations and with adjustments for several maternal (age at delivery, smoking habit, and alcohol intake) and child-related (sex and prevalence of other congenital diseases) variables.

**Results:**

The prevalence of self-harm ideation in 238 mothers of infants with CL/P at 1 and 6 months were 14.7% (35/238) and 18.8% (45/238) [8.2% (8,185/100,062) and 12.9% (12,875/100,062) in the control group], respectively. The odds ratio (95% confidence interval) for maternal self-harm ideation increased with CL/P prevalence [1.80 (1.22–2.65) and 1.47 (0.98–2.18)] at 1 and 6 months of age, respectively. After stratified by the prevalence of cleft lip, we found significant differences in the CL ± P group but not in the CP group. Furthermore, persistent self-harming ideation was associated with a higher risk in the CL ± P group [2.36 (1.43–3.89)].

**Conclusion:**

CL/P, particularly cleft lip, which is more noticeable externally, was associated with an increased prevalence of maternal self-harm ideation. The findings in this study indicate some potential benefits of increasing support for mothers who have infants with CL/P.

## Introduction

Orofacial cleft defects, such as cleft lip and/or cleft palate (CL/P), are among the most common birth defects, with a prevalence of approximately 1 in 500–700 births (1). Based on the patterns of fusion failure during craniofacial embryogenesis, CL/P is categorized into cleft lip with (CL + P) or without cleft palate (CL), and isolated cleft palate (CP) (1, 2). Previous studies have inconsistent findings regarding the prevalence of CL/P (3, 4); however, most agree that the condition negatively affects the psychological state of both children and their parents (5–8). In a recent study using a dataset from the Japan Environment and Children's Study (JECS), a nationwide prospective survey of children's health in Japan, Sato et al. (8) reported an increased risk of postpartum depression in mothers of infants with CL/P. However, the impacts of the prevalence of CL/P on maternal postpartum depression were inconsistent throughout the survey period.

Maternal postpartum depression, reported by a meta-analysis study, is a common mood disorder experienced by 13%–19% of mothers within the first 12 months postpartum (9). Poor support and care for maternal postpartum depression affect the mother and may cause adverse effects to infants as well, such as child abuse or maternal neglect. Van Horne et al. discovered a higher prevalence of maltreatment (cumulative probability of 7.62%) in children with CL/P than in children with other congenital diseases such as Down syndrome and spina bifida (approximately 5%) (10, 11). In our previous study, we reported an increased risk of maternal bonding difficulties in mothers of infants with CL/P (6).

Suicide resulting from postpartum depression has been reported to be a leading cause of maternal death after childbirth (12–14). The Edinburgh Postpartum Depression Scale (EPDS), a screening tool to identify parental postpartum depression (defined as a total score of ≥9), includes item #10, which assesses parental self-harm ideation (15). When assigning a score to item #10 (≥1), the EPDS guidelines strongly recommend immediate further evaluation and risk assessment for the mother to ensure maternal and infant safety (13, 15–18). New mothers with self-harm ideation have been reported to also experience higher long-term psychiatric morbidity (16). Schiff and Grossman (17) revealed an increased risk of infant death in the first year of life among new mothers hospitalized for a suicide attempt. Thus, screening and assessing self-harm ideation and maternal postpartum depression in new mothers have received considerable attention in recent years. However, the association between children with CL/P and maternal self-harm ideation remains unknown.

As has been suggested by Scheller et al. (7), CL/P in newborns, particularly CL, may have a high impact on maternal affection, as it significantly affects external appearance. This notion has been debated in some previous reports (4, 8, 19); however, this association remains unclear. Thus, we conducted a secondary analysis using a nationwide dataset from the JECS to investigate the impact of CL/P, particularly the presence of cleft lip (CL+/−P), on self-harm ideation in new mothers.

## Methods

### Data collection and participants

**Figure 1:**
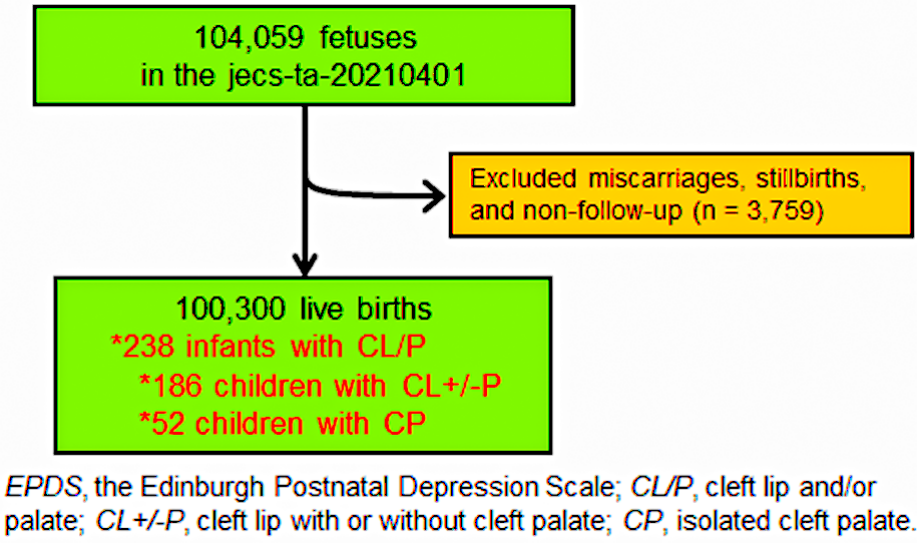
Flow chart detailing the recruitment process for study participants.

This prospective cohort study was conducted according to the guidelines of the Declaration of Helsinki of 1975 (2008 revision). We used anonymized data from the jecs-ta-20190930-qsn and jecs-qa-20210401 datasets, released in October 2019 and April 2021, respectively. The JECS protocol, which has been published elsewhere (20, 21), was reviewed and approved by the Ministry of the Environment's Institutional Review Board on Epidemiological Studies (no. 100406001) and the Ethics Committees of all participating institutions (no. 100910001). The aim and procedure of the study were explained to all pregnant women in early pregnancy stages who visited the co-operating healthcare providers in hospitals or local government offices for their first prenatal examinations between January 2011 and March 2014. Written informed consent (78.5% consent rate) was obtained from all participants, who then completed self-administered and medical record-transfer questionnaires. Subsequently, they underwent medical assessments by physicians, midwives, nurses, and/or research coordinators at delivery and 1 month later. Details regarding data processing, validation, and verification of the perinatal assessment have been previously described (20–22). We enrolled 104,059 pregnancies from 15 regional centers in the JECS. Of these, 3,759 (3.6%) were miscarriages or stillbirths, or the participants’ mothers were lost to follow-up. Therefore, the final sample consisted of 100,300 live births (Figure 1).

### Prevalence of orofacial cleft defects (exposure measure)

We employed a secondary data analysis approach using the JECS dataset. The data on CL/P and other congenital anomalies were extracted from medical record transcripts at delivery and 1 month later, on JECS transcription forms (6, 23, 24). A check-box for each CL/P type (CL, CLP, and CP) was included in the transcription form, and medical professionals marked the corresponding box when any orofacial cleft defect of interest was observed. In addition to CL/P prevalence, and in order to examine the influence of a more visible congenital defect on maternal self-harm ideation, the mothers of infants with CL/P were divided into two groups: (1) CL+/−P (mothers of infants with CL with or without CP) and (2) CP (mothers of infants with isolated cleft palate), as a control group with less visible issues.

### Self-harm ideation (outcome measure)

The EPDS, a 10-item psychological rating scale for evaluating postpartum depression that was established by Cox et al. (15), was completed by participating mothers at their 1-month check-ups following delivery. To focus on the association between maternal self-harm ideation and children with CL/P, we performed an analysis based on the responses to item #10 in the EPDS. The item #10 question in the EPDS was: “In the past 7 days, the thought of harming myself has occurred to me?” Participants chose from the following four response options: “Yes, quite often,” “Sometimes,” “Hardly ever,” and “Never” (15, 18). To ensure mother-infant safety when indicating an affirmative response to item #10, participating mothers were categorized into the reference (Never) and self-harm ideation (all other responses) groups. Furthermore, some participants were also classified into a chronic group based on affirmative responses regarding self-harm ideation at both 1 and 6 months following delivery.

### Covariates

The design of the questionnaire has been described in detail in previous reports (20, 22, 25). Sociodemographic characteristics (maternal age, parity status, educational attainment, child's sex, and household income), lifestyle (maternal drinking and smoking habits and partner support for childcare), and health status variables (maternal history of depression and prevalence of other congenital diseases in children), which have been identified as risk factors for postpartum depression or postpartum suicide, were included in the logistic regression models as covariates (8, 22, 26). Information such as maternal age at delivery, parity status, infant sex, and the prevalence of other congenital diseases (except CL/P) was retrieved from the patients’ medical records. Other variables, such as annual household income, maternal educational attainment, and smoking/drinking habits, were assessed using a self-administered questionnaire taken by the mothers during pregnancy. Partner support for childcare was also assessed using a follow-up questionnaire at 1 month postpartum.

Using these data, participants were categorized into different groups based on the following variables: child's sex (male or female); parity status (primipara or multipara); annual household income in Japanese yen (<2 million, 2–4 million, 4–6 million, or ≥6 million yen); maternal educational attainment (junior or high school, junior college [technical or junior college], or university [university or graduate]); maternal smoking history [never, stopped smoking before or during pregnancy (previously did but quit before realizing current pregnancy, previously did but quit after realizing current pregnancy), currently smoking]; maternal alcohol intake [never, stopped drinking before or during pregnancy (previously did but quit before realizing current pregnancy, previously did but quit after realizing current pregnancy), or current drinker]; partner support for childcare (yes [satisfied or somewhat satisfied] or no [somewhat unsatisfied or unsatisfied]); maternal history of depression before pregnancy (absence or presence); and other congenital disease in infants (absence or presence).

### Statistical analysis

Maternal age at delivery was presented as median and interquartile range, and categorical variables were presented as numbers and percentages (Table 1). Categorical variables were presented as numbers and percentages. The “missing at random” assumption was applied for any missing data, and multiple imputations were used with the multivariate normal imputation method (27). An imputation model that included all variables used in the main analysis was independently applied to 10 copies of the data, each containing suitably imputed missing values. As per Rubin's rules, the imputed values of the variables were estimated using means and adjusted standard errors obtained from the observed data (28). The missing values according to variables are summarized in Supplementary Table S1.

**Table 1:** Baseline characteristics of *n* = 100,300 mother-infant pairs that participated in the JECS.

	Maternal self-harm ideations, *n* (%)			
	1 month postpartum		6 months postpartum	
	Absence	Presence	Absence	Presence
Characteristics	92,080 (91.8)	8,220 (8.2)	87,380 (87.1)	12,920 (12.9)
Age at delivery, median (IQR)				
	31 (28, 35)	31 (28, 35)	31 (28, 35)	31 (28, 35)
Children with or without facial cleft, *n* (%)				
Healthy	91,877 (91.8)	8,185 (8.2)	87,187 (87.1)	12,875 (12.9)
CL+/−P	155 (83.5)	31 (16.5)	148 (79.5)	38 (20.5)
CP	48 (91.7)	4 (8.3)	45 (87.1)	7 (12.9)
Child sex				
Male	47,153 (91.7)	4,249 (8.3)	44,731 (87.0)	6,671 (13.0)
Female	44,927 (91.9)	3,971 (8.1)	42,649 (87.2)	6,249 (12.8)
Parity status				
Primiparae	36,782 (90.3)	3,929 (9.7)	35,476 (87.1)	5,235 (12.9)
Multiparae	55,298 (92.8)	4,291 (7.2)	51,904 (87.1)	7,685 (12.9)
Household income (million yen/year)				
<2	5,089 (83.7)	992 (16.3)	4,632 (76.2)	1,449 (23.8)
2 to <4	31,135 (90.2)	3,376 (9.8)	29,229 (84.7)	5,282 (15.3)
4 to <6	31,310 (93.0)	2,369 (7.0)	29,859 (88.7)	3,820 (11.3)
≥6	24,546 (94.3)	1,483 (5.7)	23,660 (90.9)	2,369 (9.1)
Educational attainment				
High school or less	32,477 (88.9)	4,056 (11.1)	30,381 (83.2)	6,152 (16.8)
Junior college	39,228 (93.2)	2,868 (6.8)	37,455 (89.0)	4,641 (11.0)
University or higher	20,375 (94.0)	1,296 (6.0)	19,544 (90.2)	2,127 (9.8)
Smoking habit				
Never	54,209 (93.1)	3,992 (6.9)	51,746 (88.9)	6,455 (11.1)
Stopped	33,728 (90.6)	3,490 (9.4)	31,803 (85.4)	5,415 (14.6)
Smoking	4,143 (84.9)	738 (15.1)	3,831 (78.5)	1,050 (21.5)
Alcohol intake				
Never	31,955 (92.2)	2,709 (7.8)	30,401 (87.7)	4,263 (12.3)
Stopped	50,937 (91.4)	4,796 (8.6)	48,249 (86.6)	7,484 (13.4)
Drinking	9,188 (92.8)	715 (7.2)	8,730 (88.2)	1,173 (11.8)
Partner support				
Yes	85,777 (92.2)	7,233 (7.8)	81,642 (87.8)	11,368 (12.2)
No	6,303 (86.5)	987 (13.5)	5,738 (78.7)	1,552 (21.3)
Maternal history of depression				
Absence	89,856 (92.4)	7,400 (7.6)	85,403 (87.8)	11,853 (12.2)
Presence	2,224 (73.1)	820 (26.9)	1,977 (65.0)	1,067 (35.0)
Other congenital diseases				
Absence	80,394 (91.9)	7,062 (8.1)	76,295 (87.2)	11,161 (12.8)
Presence	11,686 (91.0)	1,158 (9.0)	11,085 (86.3)	1,759 (13.7)

JECS, Japan Environment and Children's Study; IQR, interquartile range; CL+/−P, cleft lip with or without cleft palate; CP, isolated cleft palate.

Using the “Healthy” group as the reference, crude and multivariate logistic regression analyses were conducted using the hierarchical multiple regression model for potential covariates, in order to estimate the odds ratios (ORs) of the prevalence of CL/P and CL+/−P or CP in children as the exposure (independent) variables for maternal self-harm ideation as the outcome (dependent) variable. For crude or adjusted analysis using the aforementioned covariates, Model 1 was analyzed after adjustments for maternal age at delivery, parity status, and sex of the child. In addition to the variables in Model 1, Model 2 included maternal factors (educational attainment, smoking and drinking habits, and history of depression), partner support, household income, and prevalence of other congenital diseases in the children (Tables 2, 3). All statistical analyses were performed using SPSS (version 24.0; IBM Corp., Armonk, NY, USA). Statistical significance was set at *p* < 0.05.

**Table 2:** Association between maternal self-harm ideation and children with orofacial cleft defects.

	Presence, *n* (%)	Crude	*p*-value	Model 1^a^	*p*-value	Model 2^b^	*p*-value
At 1 month postpartum							
Healthy	8,185 (8.2)	Ref		Ref		Ref	
CL/P	35 (14.7)	1.93 (1.32–2.81)	0.001	1.92 (1.32–2.80)	0.001	1.80 (1.22–2.65)	0.003
CL+/−P	31 (16.5)	2.21 (1.47–3.32)	<0.001	2.21 (1.47–3.32)	<0.001	2.09 (1.37–3.18)	0.001
CP	4 (8.3)	1.01 (0.36–2.80)	0.990	0.99 (0.36–2.76)	0.987	0.87 (0.30–2.49)	0.795
At 6 months postpartum							
Healthy	12,875 (12.9)	Ref		Ref		Ref	
CL/P	45 (18.8)	1.57 (1.07–2.29)	0.020	1.56 (1.06–2.28)	0.023	1.47 (0.98–2.18)	0.060
CL+/−P	38 (20.5)	1.74 (1.15–2.63)	0.009	1.72 (1.14–2.61)	0.010	1.63 (1.06–2.51)	0.026
CP	7 (12.9)	1.00 (0.43–2.33)	0.995	1.01 (0.43–2.36)	0.990	0.91 (0.38–2.20)	0.836

CL/P, cleft lip and/or palate; CL+/−P, cleft lip with or without cleft palate; CP, isolated cleft palate.

Crude and multivariate logistic regression analyses regarding the prevalence of CL/P or presence/absence of cleft lip (CL+/−P or CP) in children were independently conducted using the “Healthy” group as the reference.

Odds ratio (95% confidence interval) (all such values) for maternal self-harm ideation.

^a^ Adjusted for maternal age, parity status, and child's sex.

^b^ Additionally adjusted for maternal factors (educational attainment, smoking and drinking habit, and history of depression), partner support, household income, and prevalence of other congenital diseases in children with Model 1.

**Table 3:** Association between persistent maternal self-harm ideation and children with orofacial cleft defects.

	Presence (%)	Crude	*p*-value	Model 1^a^	*p*-value	Model 2^b^	*p*-value
Healthy	4,802 (4.8)	Ref		Ref		Ref	
CL+/−P	21 (11.3)	2.53 (1.57–4.09)	<0.001	2.52 (1.56–4.08)	<0.001	2.36 (1.43–3.89)	0.001
CP	2 (4.0)	0.83 (0.20–3.42)	0.794	0.83 (0.20–3.42)	0.791	0.69 (0.16–2.91)	0.609

CL+/−P, cleft lip with or without cleft palate; CP, isolated cleft palate.

Odds ratio (95% confidence interval) (all such values).

^a^ Adjusted for maternal age, parity status, and child's sex.

^b^ Additionally adjusted for maternal factors (educational attainment, smoking and drinking habit, and history of depression), partner support, household income, and prevalence of other congenital diseases in children with Model 1.

## Results

The study participants’ characteristics are presented in Table 1. Of the 238 infants who had orofacial cleft defects (overall prevalence, 0.24%), 114 children with CL/P (47.9%), 72 with CL (30.3%), and 52 with CP (21.8%) were included in the analysis. Maternal self-harm ideation was reported by 8,220 participants (8.2%) at 1 month and 12,920 participants (12.9%) at 6 months postpartum. Of these, 4,825 (4.8%) confirmed self-harm ideation at both 1 and 6 months postpartum. The mean (standard deviation) EPDS scores at 1 and 6 months postpartum in participants without self-harm ideation were 4.5 (2.8) and 3.9 (2.6), respectively. The corresponding scores for participants with self-harm ideation were 11.6 (4.3) and 9.9 (4.1), respectively. The percentages of participants with EPDS scores above the cut-off (≥9) at 1 and 6 months postpartum were 2.2% and 5.9% in participants without self-harm ideation and 43.2% and 63.5% in participants with self-harm ideation, respectively. Regarding the tendency toward self-harm ideation among the three CL/P groups, the proportion of participants who reported self-harm ideation in the CL+/−P group was the highest at each time point (Table 1) and was persistent (Supplementary Table S2).

The crude and adjusted ORs [95% confidence interval (CI)] of the CL/P group for maternal self-harm ideation among mothers at 1 and 6 months postpartum are presented in Table 2. In model 2, including all covariates, the adjusted ORs for maternal self-harm ideation at 1 and 6 months postpartum were 1.80 (1.22–2.65, *p* = 0.003) and 1.47 (0.98–2.18, *p* = 0.060), respectively (Table 2). Similarly, after dividing the CL/P group into the CL+/−P and CP groups, we calculated the crude and adjusted ORs of the CL+/−P associated with maternal self-harm ideation, as presented in Table 2. After adjusting for all covariates, the adjusted ORs for maternal self-harm ideation at 1 and 6 months postpartum were 2.09 (1.37–3.18, *p* = 0.001) and 1.63 (1.06–2.51, *p* = 0.026) in the CL+/−P group and 0.87 (0.30–2.49, *p* = 0.795) and 0.91 (0.38–2.20, *p* = 0.836) in the CP group, respectively (Table 2).

When the participants were categorized based on maternal self-harm ideation at both 1 and 6 months postpartum as the outcome measure, the adjusted ORs for persistent self-harm ideation in model 2 were greater in participating mothers of infants with CL+/−P [2.36 (1.43–3.89), *p* = 0.001], but not CP [0.69 (0.16–2.91), *p* = 0.609; Table 3]. Regarding the association between maternal self-harm ideation and the overall prevalence of orofacial CL/P, the adjusted OR was 1.95 (1.22–3.13, *p* = 0.006).

## Discussion

The key findings of this study which included a nationwide dataset from a prospective birth cohort study in Japan, are that CL/P, particularly the prevalence of CL (CL+/−P), was critically associated with self-harm ideation in new mothers during the postpartum period. The results of this study provide a call to action for multidisciplinary cleft care providers with regard to supporting mothers who have infants with CL/P.

Congenital diseases such as heart defects or CL/P cause distress and anxiety in parents (8, 29). Furthermore, the visual impact of CL/P may influence maternal-infant bonding owing to even greater distress and anxiety; however, this notion remains unproven (5–7). Our findings reveal the potentially unfavorable impact of CL/P, particularly in cases where the condition is more obvious externally, on maternal self-harm ideation. According to Sato et al. (8), the prevalence of postpartum depression in mothers of infants with CL/P in the early postpartum period was inconsistent, specifically in the CL + P group at 1 month postpartum, as well as in the CP group at 6 months postpartum. In contrast, the association between maternal self-harm ideation and CL/P was representative at both 1 and 6 months postpartum. Similarly, in the CL+/−P group, postpartum mothers consistently and persistently thought of self-harm, compared with the isolated CP group (Tables 2, 3). As has been observed in children with accidental injuries or burns (30–32), orofacial cleft defects, particularly the external appearance of CL puts parents at risk for developing substantial post-traumatic stress symptoms owing to guilty feelings and anxiety (33).

In addition to higher maltreatment rates in children with CL/P (10, 11), although specific causalities were not identified, the mortality rate of patients with CL/P has been reported to be high (34, 35). The intensity and duration of maternal postpartum depression can interfere with a child's socioemotional and behavioral development (36, 37). Self-harm ideation, often observed in severe depression potentially prolongs the comorbidity of depression (16). Our recent study revealed significant neurodevelopmental delays in children with CL/P that spanned a broad range of domains, such as communication, problem-solving, and personal-social behaviors (24). Thus, severe maternal postpartum depression with self-harm ideation owing to the birth prevalence of orofacial cleft defects can have lasting adverse impacts on the cognitive, socioemotional, and behavioral development of children with CL/P. Understanding the impact of visible congenital differences, such orofacial cleft defects, on maternal self-harm ideation and child safety, growth, and development may help prevent future issues.

Additionally, since the surgical reconstruction of CL is mostly performed around 3 months of age (38), the external visibility of CL should be markedly improved at 6 months postpartum. However, the prevalence of maternal self-harm ideation in the CL+/−P group increased from 1 to 6 months postpartum, similar to what was observed in the control group. Information on surgical histories for CL/P treatment and care was not included in this dataset; thus, it remains unknown whether early surgical intervention performed on CL may have contributed to recovery from self-harm ideation in some of the participating mothers. Future studies are warranted to clarify the effectiveness of multidisciplinary intervention for children with CL/P.

### Strengths and limitations

This study had several strengths and limitations. The JECS dataset, covering approximately 45% of infants born in Japan from 2011 to 2014, provided population-based control participants with more statistical power (20). However, the relatively low number of children with CL/P was a study limitation. In addition, the data collection methods in the JECS did not include a query regarding antenatal diagnosis and/or screening. Johns et al. (39) suggested that antenatal diagnosis decreases maternal depressive symptoms in mothers of infants with CL/P. Thus, our findings cannot be generalized to all mothers of children with CL/P. Finally, the underlying mechanism behind the increased risk of self-harm ideation due to CL rather than CP remains unknown. To clarify causality, future studies with more appropriate designs are warranted.

## Conclusion

CL/P, particularly CL, critically impacts and increases the prevalence of self-harm ideation in new mothers during the postpartum period.

## Data Availability

The data analyzed in this study is subject to the following licenses/restrictions: Data are unsuitable for public deposition owing to ethical restrictions and the legal framework of Japan. It is prohibited by the Act on the Protection of Personal Information (Act No. 57 of May 30, 2003, amended on September 9, 2015) to publicly deposit data containing personal information. Ethical Guidelines for Medical and Health Research Involving Human Subjects enforced by the Japan Ministry of Education, Culture, Sports, Science and Technology and the Ministry of Health, Labour and Welfare also restrict the open sharing of epidemiological data. Requests to access these datasets should be directed to all inquiries about access to data were sent to jecs-en@nies.go.jp. The person responsible for handling inquiries sent to this e-mail address is Dr. Shoji F. Nakayama, JECS Program Office, National Institute for Environmental Studies.

## Group members of Japan Environment and Children's Study

Menbers of the JECS Group as of 2022: Michihiro Kamijima (principal investigator, Nagoya City University, Nagoya, Japan), Shin Yamazaki (National Institute for Environmental Studies, Tsukuba, Japan), Yukihiro Ohya (National Center for Child Health and Development, Tokyo, Japan), Reiko Kishi (Hokkaido University, Sapporo, Japan), Nobuo Yaegashi (Tohoku University, Sendai, Japan), Koichi Hashimoto (Fukushima Medical University, Fukushima, Japan), Chisato Mori (Chiba University, Chiba, Japan), Shuichi Ito (Yokohama City University, Yokohama, Japan), Zentaro Yamagata (University of Yamanashi, Chuo, Japan), Hidekuni Inadera (University of Toyama, Toyama, Japan), Takeo Nakayama (Kyoto University, Kyoto, Japan), Tomotaka Sobue (Osaka University, Suita, Japan), Masayuki Shima (Hyogo Medical University, Nishinomiya, Japan), Hiroshige Nakamura(Tottori University, Yonago, Japan), Narufumi Suganuma (Kochi University, Nankoku, Japan), Koichi Kusuhara (University of Occupational and Environmental Health, Kitakyushu, Japan), and Takahiko Katoh (Kumamoto University, Kumamoto, Japan).
