# Supplementary material for: Risk of self-harm ideation in mothers of children with orofacial cleft defects: the Japan environment and children's study
Source: Front Glob Womens Health. 2024 Sep 17;5:1302808. doi: 10.3389/fgwh.2024.1302808 (PMC11457732; doi:10.3389/fgwh.2024.1302808)
Supplement: Supplementary file 1 [file Table1.docx]

Table S1 Categorical characteristics of missing data in the JECS.

| **Missing (n [%])** | **Maternal self-harm ideations, n (%)** | | |
| --- | --- | --- | --- |
|  | **Control** | **CL±P** | **CP** |
| **Age at delivery** | 8 (0.0) | 0 (0.0) | 0 (0.0) |
| **1 month postpartum** | 2,496 (2.5) | 21 (11.3) | 1 (1.9) |
| **6 months postpartum** | 7,166 (7.2) | 29 (15.6) | 4 (7.7) |
| **Child sex** | 18 (0.2) | 0 (0.0) | 0 (0.0) |
| **Parity status** | 2,410 (2.4) | 7 (3.8) | 2 (3.8) |
| **Household income (million yen/ year)** | 8,771 (8.8) | 24 (12.9) | 1 (1.9) |
| **Educational attainment** | 2,316 (2.3) | 9 (4.8) | 0 (0.0) |
| **Smoking habit** | 986 (1.0) | 3 (1.6) | 0 (0.0) |
| **Alcohol intake** | 853 (0.9) | 3 (1.6) | 0 (0.0) |
| **Partner support** | 15,531 (15,5) | 50 (26.9) | 8 (15.4) |
| **Maternal history of depression** | 1,278 (1.3) | 5 (2.7) | 0 (0.0) |
| **Other congenital disease** | 0 (0.0) | 0 (0.0) | 0 (0.0) |
