# Supplementary material for: Risk of self-harm ideation in mothers of children with orofacial cleft defects: the Japan environment and children's study
Source: Front Glob Womens Health. 2024 Sep 17;5:1302808. doi: 10.3389/fgwh.2024.1302808 (PMC11457732; doi:10.3389/fgwh.2024.1302808)
Supplement: Supplementary file 2 [file Table2.docx]

| **Characteristics** | | **Chronic self-harm ideations in mothers, n (%)** | | |
| --- | --- | --- | --- | --- |
|  |  | **Absence, n (%)** | | **Presence, n (%)** |
|  |  | **95,475 (95.2)** | | **4,825 (4.8)** |
| **Age at delivery, Median (IQR)** | | | | |
|  | 31 (28, 35) | | 31 (28, 35) | |
| **Children with CL+/-P or CP, n (%)** | | | | |
| Healthy | 95,260 (95.2) | | 4,802 (4.8) | |
| CL+/-P | 165 (88.7) | | 21 (11.3) | |
| CP | 50 (96.0) | | 2 (4.0) | |
| **Child sex** | | | | |
| Male | 48,921 (95.2) | | 2,481 (4.8) | |
| Female | 46,554 (95.2) | | 2,344 (4.8) | |
| **Parity status** | | | | |
| primiparae | 38,584 (94.8) | | 2,127 (5.2) | |
| multiparae | 56,891 (95.5) | | 2,699 (4.5) | |
| **Household income (million yen/ year)** | | | | |
| < 2 | 5,402 (88.8) | | 680 (11.2) | |
| 2 to < 4 | 32,460 (94.1) | | 2,050 (5.9) | |
| 4 to < 6 | 32,350 (96.1) | | 1,329 (3.9) | |
| ≥ 6 | 25,263 (97.1) | | 766 (2.9) | |
| **Educational attainment** | | | | |
| High school or less | 33,991 (93.0) | | 2,541 (7.0) | |
| Junior college | 40,492 (96.2) | | 1,605 (3.8) | |
| University or higher | 20,992 (96.9) | | 679 (3.1) | |
| **Smoking habit** | | | | |
| Never | 55,952 (96.1) | | 2,250 (3.9) | |
| Stopped | 35,119 (94.4) | | 2,099 (5.6) | |
| Smoking | 4,404 (90.2) | | 477 (9.8) | |
| **Alcohol intake** | | | | |
| Never | 33,108 (95.5) | | 1,557 (4.5) | |
| Stopped | 52,903 (94.9) | | 2,831 (5.1) | |
| Drinking | 9,464 (95.6) | | 438 (4.4) | |
| **Partner support** | | | | |
| Yes | 88,864 (95.5) | | 4,146 (4.5) | |
| No | 6,611 (90.7) | | 680 (9.3) | |
| **History of depression** | | | | |
| Absence | 93,041 (95.7) | | 4,215 (4.3) | |
| Presence | 2,433 (79.9) | | 611 (20.1) | |
| **Other congenital diseases** | | | | |
| Absence | 83,325 (95.3) | | 4,131 (4.7) | |
| Presence | 12,149 (94.6) | | 695 (5.4) | |
| Abbreviations: JECS = Japan Environment and Children’s Study; IQR = interquartile range;  CL+/-P = cleft lip with or without cleft palate; CP = isolated cleft palate. | | | | |

**TABLE S2　Baseline characteristics of n=100,300 mother-infant pairs.**
